# Absence of first‐pass isolation is associated with poor pulmonary vein isolation durability and atrial fibrillation ablation outcomes

**DOI:** 10.1002/joa3.12629

**Published:** 2021-09-06

**Authors:** Yuichi Ninomiya, Koichi Inoue, Nobuaki Tanaka, Masato Okada, Koji Tanaka, Toshinari Onishi, Yuko Hirao, Takafumi Oka, Hiroyuki Inoue, Kohtaro Takayasu, Ryo Nakamaru, Ryo Kitagaki, Yasushi Koyama, Atsunori Okamura, Katsuomi Iwakura, Mitsuru Ohishi, Kenshi Fujii

**Affiliations:** ^1^ Cardiovascular Center Sakurabashi Watanabe Hospital Osaka Japan; ^2^ Department of Cardiovascular Medicine and Hypertension Kagoshima University Graduate School of Medical and Dental Sciences Kagoshima Japan

**Keywords:** adenosine triphosphate, atrial fibrillation, durable pulmonary vein isolation, first‐pass isolation, pulmonary vein reconnection

## Abstract

**Background:**

Pulmonary vein (PV) reconnection is the main cause of atrial fibrillation (AF) recurrence. This study aimed to examine the effect of first‐pass PV isolation (PVI) on PV reconnection frequency during the procedure and on AF ablation outcomes.

**Methods:**

This retrospective study included 446 patients with drug‐refractory AF (370 men, aged 64 ± 10 years) who underwent initial PVI using an open‐irrigated contact force catheter between January 2015 and October 2016. We investigated the effect of first‐pass PVI on PV reconnection during spontaneous PV reconnection and dormant conduction after an adenosine triphosphate challenge.

**Results:**

First‐pass PVI was achieved in 69% (617/892) of ipsilateral PVs, of which we observed PV reconnection during the procedure in 134 (22%) PVs. This value was significantly lower than that observed in those without first‐pass PVI (50%, 138/275) (*P* < .0001). We divided the subjects into two groups based on the presence or absence of first‐pass PVI in at least one of two ipsilateral PVs: first‐pass (n = 383, 86%) and non‐first‐pass groups (n = 63, 14%). The 2‐year AF recurrence‐free rate was significantly higher in the first‐pass group than in the other group (75% vs 59%, log‐rank *P* = .032). In 78 patients with repeat AF ablation, the PV reconnection rate in the second procedure was significantly lower in PVs that had first‐pass isolation in the first procedure (34% vs 73%, *P* < .0001).

**Conclusions:**

Absence of first‐pass PVI was associated with a higher frequency of spontaneous PV reconnection and dormant conduction and poor ablation outcomes. First‐pass isolation may be a useful marker for better PVI durability.

## Graphical abstract

The 2‐year AF recurrence‐free rate was significantly higher in the first‐pass group than in the other group (75% vs. 59%, log‐rank P = 0.032). Absence of first‐pass PVI was associated with a higher frequency of spontaneous PV reconnection and dormant conduction and poor ablation outcomes. First‐pass isolation may be a useful marker for better PVI durability.

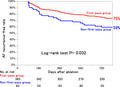

## 1 INTRODUCTION

Pulmonary vein (PV) isolation is the basis for atrial fibrillation (AF) ablation.^1^, ^2^ As isolated PV reconnection is the most often reported cause of recurrent AF,^3^, ^4^ the achievement of durable PV isolation (PVI) is vital in reducing the AF recurrence rate. Transient PV reconnection (dormant conduction, DC) that is induced by adenosine triphosphate (ATP) is an indicator of poor PV durability.^5^ We previously investigated the usefulness of VISITAG‐guided AF ablation^6^ by comparing the AF ablation outcomes between the VISITAG‐guided AF ablation group and contact force (CF)‐guided AF ablation group without an automated annotation algorithm. First‐pass PVI, which was defined as the achievement of successful PVI before initial circle completion, was observed more frequently in the VISITAG‐guided AF ablation group than in the CF‐guided AF ablation group. The 1‐year success rate without anti‐arrhythmic drug (AAD) use was higher in the VISITAG‐guided AF ablation group than in the CF‐guided AF ablation group. Based on our results, we hypothesized that first‐pass PVI may be associated with PVI durability and good AF ablation outcomes. However, few studies have focused on the association between first‐pass PVI and PVI durability, as well as AF ablation outcomes. Thus, this study aimed to examine the effect of first‐pass PVI on the frequency of PV reconnection during the procedure and on AF ablation outcomes.

## 2 METHODS

### 2.1 Study population

This retrospective study included 446 patients with drug‐refractory AF (370 men, age 64 ± 10 years) between January 2015 and October 2016; of these patients, 191 had paroxysmal AF and an initial PVI with an open‐irrigated CF catheter for AF. ATP challenge tests during the procedure were performed at the discretion of attending doctors. A total of 82 patients who did not undergo ATP challenge tests were excluded. We divided the subjects into two groups according to their body mass index (BMI) based on the definition proposed by the Japan Society of Obesity. Those in the obesity group had BMI ≥25 kg/m^2^. Others were assigned to the normal‐weight group.

This study was approved by the Ethics Committee of our hospital. Written informed consent for AF ablation was obtained from all patients. All AADs were discontinued for at least five half‐lives prior to study entry.

### 2.2 Multi‐detector computed tomography (CT) of the left atrium (LA)

All patients underwent 3D reconstruction of the LA and PV on a 64‐slice multi‐detector CT scanner using a 160‐detector low dynamic volume scanner within 1 week prior to ablation therapy (Brilliance CT 64, Phillips Medical Systems).

### 2.3 Electrophysiological studies

Electrophysiological studies were performed under shallow sedation with a bolus dose of pentazocine combined with a continuous dose of dexmedetomidine hydrochloride. Esophageal temperature monitoring (SensiTherm, St. Jude Medical) was performed, and respiration was controlled by positive pressure ventilation in all patients. Surface and intracardiac electrocardiograms were digitally recorded and stored (LabSystem™ PRO EP Recording System, Bard Clearsign™, Boston Scientific Corporation). Bipolar electrograms were filtered from 30 to 500 Hz. A 6‐Fr, 20‐pole, 3‐site mapping catheter (BeeAT, Japan Lifeline) was placed from the right brachial vein or jugular vein for pacing, recording, and internal cardioversion. A 20‐pole catheter was placed in the right atrium or superior vena cava. Two long sheaths (SL0, St. Jude Medical) were introduced into the LA via the femoral vein using a single transseptal puncture technique. A single 150 IU/kg bolus of heparin was administered after the trans‐septal puncture and was repeated as necessary to maintain an activated clotting time >300 s. After the transseptal puncture, a single 150 IU/kg bolus of heparin was administered, and additional heparin doses were repeated as necessary to ensure that the activated clotting time was >300 s. In patients with sustained AF, electrical cardioversion was performed at the beginning of the procedure, and the presence or absence of immediate AF recurrence was searched for under sedation with thiamylal sodium. In those whose sinus rhythm was maintained, isoproterenol was initially administered at a 1‐2 μg/min dose without additional sedation; subsequently, the dosage was gradually increased up to a maximum of 20 μg/min while blood pressure was carefully monitored. Isoproterenol was administered for 5 min. For induced AF, electric cardioversion was performed again, and immediate AF recurrence was assessed using the aforementioned mapping technique.^7^ For AF triggers observed from the PVs, PVI of the arrhythmogenic ipsilateral PVs was attempted first, as the waiting time after PVI of arrhythmogenic ipsilateral PVs may be longer than that after PVI of the opposite ipsilateral PVs.

### 2.4 Integration of CARTO with CT

CT data were integrated into CARTO using SoundStar Ultrasound Catheter (Biosense Webster) (SoundMerge), as previously described.^8^ SoundMerge comprises two steps: landmark registration by a single landmark and surface registration with shells created by LA and PV posterior walls and roofs. In most cases, the best landmark is the posterior carina of the right PV, which is visualized and easily identified. If clear echo images cannot be obtained, additional points can be added using an open‐irrigated ThermoCoolSmartTouch catheter (Biosense Webster). Currently, we routinely add linear points at the roof of the left and right PVs and at the bottom of the left and right PVs using a Thermocool Smart Touch catheter.

### 2.5 Setting up an automatic annotation algorithm

Parameters of the automated ablation annotation system were set (Carto 3 System, VISITAG Module, Biosense Webster) as follows: (1) catheter stability range of motion ≤1.5 mm, (2) catheter stability duration >5 s, and (3) CF ≥5 g, time ≥25%. The lesion tag had a radius of 3 mm. The minimum value of force‐time integral (FTI) was set at 150 gs, and this level was met or exceeded at each ablation site.

### 2.6 PVI

**FIGURE 1:**
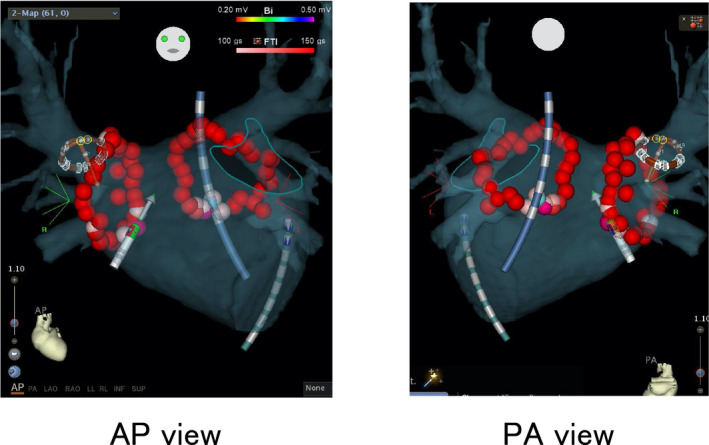
Pulmonary vein isolation performed using an automated algorithm with predefined criteria for catheter stability (range of motion <1.5 mm and duration >5 s). Ablated points are displayed in tags with a radius of 3 mm. White automated annotation tag, force‐time integral (FTI) < 100 gs; pink automated annotation tag, 100 gs < FTI < 150 gs; and red automated annotation tag, FTI > 150 gs. The tag turns red when the FTI reaches ≥150. Left panel, anteroposterior view; right panel, posteroanterior view. AP, anteroposterior; PA, posteroanterior

The VISITAG‐guided PVI was performed using an open‐irrigated ThermoCoolSmartTouch catheter (Biosense Webster) and a circular mapping catheter (Lasso, Biosense Webster; adjustable circumference, 15‐25 mm; interelectrode pacing, 1‐2 mm), as previously described.^6^ The RF energy was delivered using a dragging method and a Stockert 70 (Biosense Webster) RF generator at 25‐30 W for areas close to the esophagus and 30‐35 W for the other areas. Each lesion was ablated for >20 s, and the FTI of each lesion was >150 gs. We intended to perform high‐density ablation to achieve a tag overlap; thus, a circular line of red tags was present in the PV on the same side (Figure 1). The function to display the interlesion distance (ILD) was unavailable during the study period. Although a minimum ILD was not defined in this study, radiofrequency applications were initiated to a target ILD of 5 mm.

When a residual conduction gap was detected after the completion of the first circle, additional radiofrequency applications were employed to complete the PVI. First‐pass PVI was defined as successful PVI at or before completion of the encircling lesion set regardless of visual gaps. Thus, PVI by ablation of visual gaps was excluded in the first‐pass isolation (FPI) in this study. Regardless of successful PVI, complete encirclement was performed without any visual gaps. We excluded ipsilateral PVs from the first‐pass PVI when the carina between the superior and inferior PVs was ablated.

PV reconnection during the procedure was defined as the sum of spontaneous PV reconnections >20 min after the initial PVI and DC after an ATP challenge. To detect spontaneous PV reconnection, the circular mapping catheter was used to repeatedly check for the presence of PV potential for >20 min after the initial isolation of all four PVs. If spontaneous PV reconnection was observed, additional ablation was performed until the PV was electrically isolated. Thereafter, ATP challenge tests were performed to evaluate the DC for each PV using rapid infusion of ATP at 0.4 mg/kg body weight under back‐up pacing from the right ventricle >20 min after the initial PVI success. In cases with a spontaneous PV reconnection within 20 min, the ATP challenge was performed after the PV reconnection was electrically isolated. Dormant PV reconnection was defined as the transient or continuous re‐appearance of PV potentials accompanied by atrial depolarization. When PV conduction reappeared during the procedure, additional radiofrequency applications were performed to the earliest PV activation site identified by the circular mapping catheter until complete PV isolation was achieved. Complete PV disconnection was confirmed by a repeat ATP challenge. After DC disappearance, no additional waiting time was needed. We also attempted to ablate non‐PV premature atrial contractions, including those of the superior vena cava, triggering atrial fibrillation by continuous intravenous infusion of isoproterenol (4‐20 μg/min). Any atrial flutter or atrial tachycardia induced by rapid atrial pacing was eliminated by linear ablation of the cavotricuspid isthmus, LA roof, and mitral valve isthmus and by ablation of focal atrial tachycardia.

### 2.7 Patient follow‐up

Patients stayed in the hospital for 3 days after the ablation for continuous rhythm monitoring. Anticoagulants were administered orally the evening of the ablation day and continued for at least 3 months after the procedure, unless pericardial effusion was detected. Continuation of AADs that were prescribed before catheter ablation (CA) was encouraged. Bepridil was prescribed to nearly all patients with non‐paroxysmal AF who were not receiving AADs before CA. Discontinuation of anti‐arrhythmia medication was recommended 3 months after CA. Patients were instructed to self‐check their pulse rate and rhythm three times daily and to visit the outpatient clinic if they experience signs and symptoms of AF recurrence, such as palpitations and dyspnea. Patients were examined in an outpatient clinic at 1, 3, 6, 9, and 12 months after the procedure and every 6 months thereafter. Holter electrocardiography and transthoracic echocardiography were performed 6 months after CA. All patients with symptoms of undocumented recurrent AF were provided with an event recorder (available for 7 days) to help identify the cause of their symptoms. After ablation, a 3‐month blank period was ensured. Freedom from AF was defined as the absence of AF lasting >30 s without the use of AAD.

Patients with recurrent AF underwent a second procedure. PV reconnection rate was compared between the ipsilateral PVs with FPI and those without FPI in the first procedure.

### 2.8 Statistical analysis

All data are presented in the form of frequency (percentage) for categorical variables and mean ± standard deviation for continuous variables. Two‐sample t‐tests or Wilcoxon rank‐sum tests were used for the comparison of continuous variables, and a chi‐square or Fisher exact test was used for between‐group comparisons of the categorical variables. The Bonferroni method was employed to adjust for multiple comparisons. The incidence of non‐recurrence of AF after ablation was estimated by the Kaplan‐Meier method, and the difference was evaluated by the log‐rank test. A multivariate Cox proportional hazards model was constructed to identify independent predictors of AF recurrence after ablation. Odds ratios were considered significant when the lower limit of the 95% confidence interval (CI) was greater than 1.0. P values were adjusted for multiple comparisons using the Bonferroni method. *P* values <.05 were considered significant. The JMP version 14.3 software (SAS Institute) was used for statistical analysis.

## 3 RESULTS

### 3.1 FPI rate

PVI was successful in all patients. FPI was achieved in 317 left PVs (71%) and 300 right PVs (67%). Altogether, 234 (53%) and 149 (33%) of the 446 patients experienced first‐pass PVI in both PVs and in one PV respectively. The study population was divided into two groups based on the presence or absence of first‐pass PVI in at least one of two ipsilateral PVs: first‐pass (n = 383, 86%) and non‐first‐pass (n = 63, 14%) groups. No significant difference in age, gender, CHADS2 score, type of AF, comorbidities, and echocardiographic data between the two groups was found; however, significant differences in BMI, PV diameter, and hemoglobin levels were observed (Table 1).

**TABLE 1:** Baseline patient characteristics

	First‐pass group	Non‐first‐pass group	*P* value
	n = 383	n = 63	
Age, years	64 ± 11	63 ± 9	.64
Male, n (%)	315 (82)	55 (87)	.32
BMI, kg/m^2^	23.9 ± 3.5	25.0 ± 3.9	.02
CHADS2 score	1.0 (0.0‐2.0)	1.0 (0.0‐2.0)	.28
Type of AF, n (%)			
Paroxysmal	163 (43)	28 (44)	.71
Persistent	149 (39)	26 (41)	
Long‐standing persistent	71 (19)	9 (14)	
Comorbidities, n (%)			
Heart failure	75 (20)	13 (21)	.85
Hypertension	199 (52)	29 (46)	.38
Diabetes mellitus	71 (19)	11 (18)	.84
History of stroke	32 (8)	2 (3)	.20
Echocardiographic data			
LAD, mm	39.7 ± 5.9	39.4 ± 5.9	.77
LVDd, mm	47.5 ± 5.5	46.7 ± 4.3	.25
LV ejection fraction (Teicholz), %	63.2 ± 11.2	65.8 ± 8.7	.08
CT evaluation			
Common LPV, n (%)	18 (5)	0 (0)	.09
All PV diameter, mm	18.3 ± 2.0	18.9 ± 2.3	.03
LSPV diameter, mm	19.5 ± 3.3	20.2 ± 3.4	.12
LIPV diameter, mm	14.9 ± 2.5	15.2 ± 2.3	.49
RSPV diameter, mm	21.5 ± 3.5	21.9 ± 3.6	.36
RIPV diameter, mm	17.3 ± 2.9	18.4 ± 3.5	.03
Maximum LA volume, mL	112.9 ± 33.3	111.5 ± 32.6	.75
Laboratory data			
Hb, g/dl	14.2 ± 1.4	13.8 ± 1.5	.046
Cre, mg/dl	0.88 (0.75‐1.00)	0.90 (0.70‐1.01)	.35
CRP, mg/dl	0.06 (0.03‐0.12)	0.07 (0.02‐0.19)	.98
BNP, pg/ml	87 (43‐176)	84 (37‐181)	.84

Note

Data are expressed as mean ± standard deviation or n (%), except for the Cre, CRP, and BNP, which are expressed as median (first quartile and third quartile).

Abbreviations: BMI, body mass index; BNP, brain natriuretic peptide; Cre, creatinine; CRP, C‐reactive protein; CT, computed tomography; Hb, hemoglobin; LAD, left atrial dimension; LIPV, left inferior pulmonary vein; LPV, left pulmonary vein; LSPV, left superior pulmonary vein; LV, left ventricle; LVDd, left ventricular diastolic dimension; PV, pulmonary vein; RIPV, right inferior pulmonary vein; RSPV, right superior pulmonary vein.

### 3.2 PV reconnection frequency during the procedure

**FIGURE 2:**
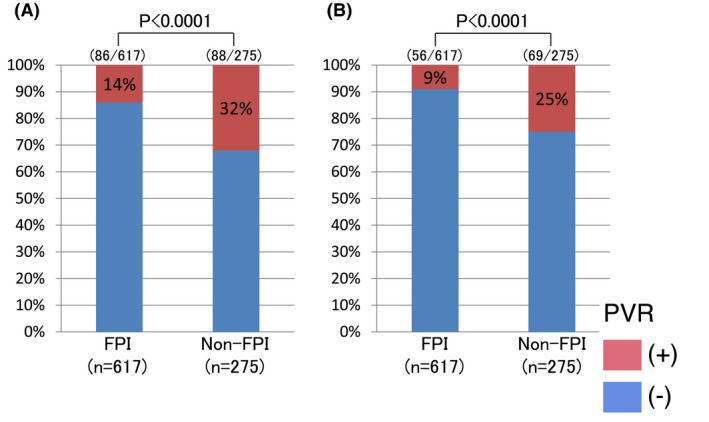
Pulmonary vein (PV) reconnection frequency during the first procedure. A, Spontaneous PV reconnection per ipsilateral PV. B, Dormant PV conduction per ipsilateral PV. FPI, first‐pass isolation; PVR, pulmonary vein reconnection

PV reconnection during the procedure was observed in 134 of 617 (22%) ipsilateral PVs with first‐pass PVI; this value was significantly lower than that in PVs without first‐pass PVI (138 of 275 [50%]) (*P* < .0001). Spontaneous PV reconnection and DC induced by ATP were observed in only 86 and 56, respectively, of 617 (14% and 9%) ipsilateral PVs with first‐pass PVI; these values were significantly lower than those observed in PVs without first‐pass PVI (88 and 69 of 275 [32% and 25%]) (*P* < .0001) (Figure 2). All spontaneous PV reconnections and DC induced by ATP challenge were successfully eliminated by additional radiofrequency application to the PV ostium.

### 3.3 First‐pass PVI and AF ablation outcomes

**FIGURE 3:**
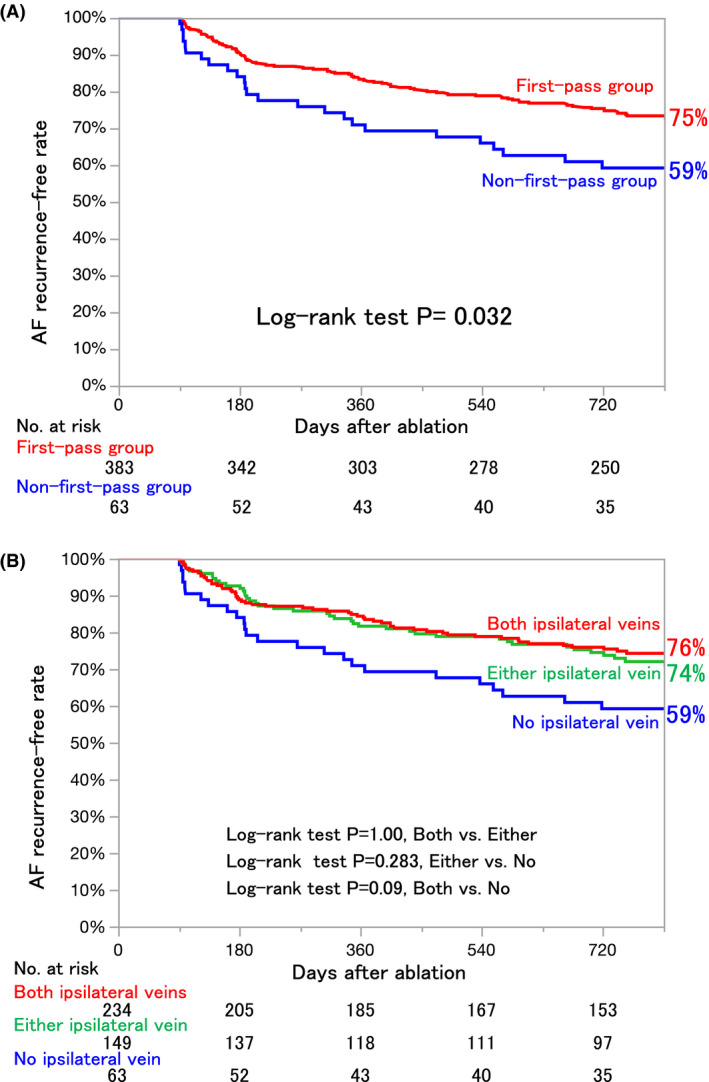
Single procedure success rate without anti‐arrhythmia drug use: Kaplan‐Meier analysis of freedom from atrial fibrillation during the follow‐up period. A, First‐pass group vs non‐first‐pass group. B, Both ipsilateral veins vs either ipsilateral vein vs no ipsilateral vein

We examined and compared the AF recurrence rates between the two groups. During the mean follow‐up period of 859 ± 211 days, 135 (30%) patients showed AF recurrence within an average of 366 ± 265 days after the procedure, whereas the remaining 311 (70%) patients were free from AF without AAD use. The 2‐year AF recurrence‐free rate was significantly higher in the first‐pass group than in the non‐first‐pass group (75% vs 59%, log‐rank *P* = .032) (Figure 3A). We also compared the AF recurrence rate among patients with FPIs in both ipsilateral PVs, those with FPIs in either ipsilateral PV, and those without FPI in both ipsilateral PVs; no significant differences among the three groups were observed (Figure 3B).

Univariate and multivariate analyses were performed to analyze the predictors of AF recurrence (Table 2). BMI have been included in the regression analysis because BMI was significantly different between the two groups, as previously described (Table 1). In the univariate Cox proportional hazards analysis, the non‐first‐pass group showed significant associations with AF recurrence (hazard ratio, 1.59; 95% CI, 1.04‐2.44; *P* = .03). Multivariate Cox proportional hazards regression analysis indicated that the absence of first‐pass PVI is an independent predictor of AF recurrence after CA (hazard ratio, 1.65; 95% CI, 1.07‐2.55; *P* = .02) after adjustment for six factors including gender, older age, and left atrial dimension.

**TABLE 2:** Cox proportional hazards regression analysis for AF recurrence

	Univariate			Multivariate		
	HR	95% CI	*P* value	HR	95% CI	*P* value
Non‐first‐pass group	1.59	1.04‐2.44	.03	1.65	1.07‐2.55	.02
PAF	0.79	0.56‐1.12	.19	0.77	0.54‐1.10	.15
Gender, male	0.97	0.63‐1.51	.91	0.92	0.59‐1.45	.73
LAD, mm	1.01	0.98‐1.04	.66	1.01	0.98‐1.04	.54
Age, years	1.00	0.98‐1.02	.99	1.00	0.98‐1.02	.91
Obesity group	0.77	0.53‐1.11	.16	0.73	0.50‐1.08	.12

Abbreviations: AF, atrial fibrillation; CI, confidence interval; HR, hazard ratio; LAD, left atrial dimension; Obesity group had a BMI of ≥25 kg/m^2^; PAF, paroxysmal AF.

### 3.4 Durability of PVI in the second procedure

**FIGURE 4:**
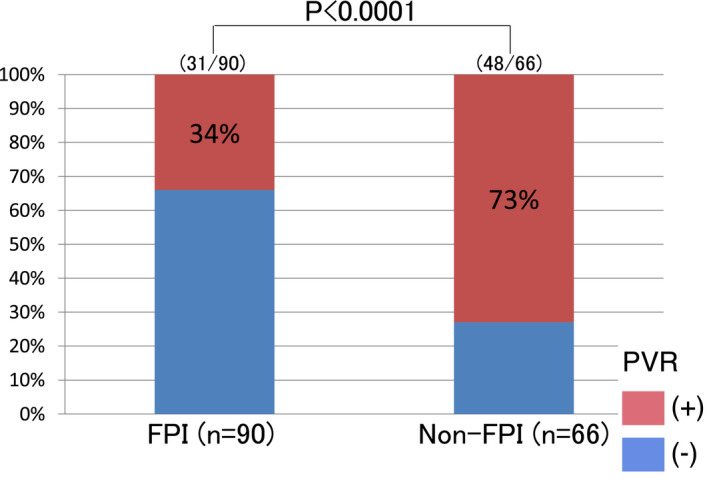
Pulmonary vein (PV) reconnection rate in the second procedure per ipsilateral PV. FPI, first‐pass isolation; PVR, pulmonary vein reconnection

A second procedure was performed in 78 of 135 patients with AF recurrence (first‐pass group, n = 58; non‐first‐pass group, n = 20). There was a significant difference in the PV reconnection rate in the second session between the FPI and non‐FPI groups (41% vs 80%, *P* < .0001). The PV reconnection rate in the second procedure was significantly lower in PVs with successful FPI in the first procedure than in others (34% vs 73%, *P* < .0001) (Figure 4).

We also investigated the association of PV reconnection observed in the first procedure with that in the second procedure. In the second procedure, the PV reconnection rate was significantly higher in PVs with reconnection during the first procedure (n = 56) than in those without (n = 100) (70% vs 40%, *P* = .0004).

## 4 DISCUSSION

The major findings of this study are as follows: (1) first‐pass PVI in both ipsilateral PVs was achieved in 234 of 446 patients (53%), (2) the prevalence of spontaneous PV reconnection and DC induced by ATP was significantly low in PVs with successful FPI, (3) the AF recurrence rate was significantly higher in the non‐first‐pass group, (4) multivariate Cox proportional hazards regression analysis indicated that the absence of first‐pass PVI is an independent predictor of AF recurrence, and (5) the PV reconnection rate in the second procedure was significantly lower in PVs with FPI in the first procedure. Moreover, the PV reconnection rate in the second procedure was significantly higher in PVs with reconnection during the first procedure, although all PV reconnections were eliminated. Our findings indicate the need for efforts toward the achievement of first‐pass PVI, which is an indicator of better PVI quality and durability. Moreover, although FPI was found to be associated with clinical outcome, it can be both a cause and a marker of favorable outcome.

In previous studies, ATP‐guided PVI showed no effect on AF ablation outcomes.^9^ However, the establishment of DC may indicate poor PVI quality. Acute PVI is achieved with reversible (tissue edema) and irreversible (tissue necrosis) atrial damage, as determined by cardiac magnetic resonance imaging.^10^ Because a certain amount of time is required for the recovery of atrial damage, the use of an ATP challenge may aid in the detection of reversible radiofrequency lesions during the procedure.

We assume that several factors inhibit FPI. The anatomical features of the regions surrounding the PV, such as the thickness of the atrial myocardium, atypical PV and LA morphology, and location of the esophagus and phrenic nerve, are likely to have a strong influence on the success of first‐pass PVI. Small PV diameter was associated with first‐pass PVI achievement in this study. A large, right superior PV was associated with a higher PV reconnection incidence in a previous study on cryoballoon ablation.^11^ In addition, lower LA voltage was reported to be associated with failure in FPI in a study of PAF.^12^ Moreover, operator skill, function of the mapping system and catheters, and difficulties associated with catheter manipulation because of an enlarged LA and unstable respiratory patterns may affect the outcomes. However, no significant difference in left atrial dimension and LA volume was found between the first‐pass and non‐first‐pass groups in this study.

We previously reported that a high mean CF reduces the ablation time but does not improve ablation results.^13^ The inability of CF monitoring to improve clinical outcomes of AF ablation may be because of the lack of information on catheter stability during energy delivery and uneven intensity of radiofrequency ablation. Hence, we demonstrated the usefulness of VISITAG‐guided AF ablation^6^ and indicated that real‐time monitoring of catheter stability and ablation intensity may improve the integrity of the lesion.

Currently, VISITAG with the ablation index (AI) is used as an alternative to FTI. The AI, which includes CF, power, and time in a weighted formula, predicts lesion depth in a canine ventricle with high accuracy. Interestingly, the reported FPI incidence using VISITAG with AI is >90%.^14^ For the achievement of FPI, VISITAG should be used with AI instead of FTI.

Monitoring PV reconnection for 20 min following initial PVI is recommended in the 2017 HRS/EHRA/ECAS/APHRS/SOLAECE expert consensus statement.^15^ However, PV reconnection was previously detected even at 60 min after the initial PVI in 10% of isolated PVs.^5^ Here, the PV reconnection rate in PVs with FPI achievement was low, and the rates of spontaneous PV reconnection and DC in the ATP challenge were 14% and 9% respectively. The PV reconnection rate further decreased with the emergence of VISITAG with AI. Thus, in PVs with first‐pass PVI, a waiting time of 20 min after the initial PVI may not be necessary. In PVs without FPI, inclusion of a waiting time may be appropriate because the PVI quality is considered poor and the PV reconnection rate is relatively high. Furthermore, in PVs without FPI, additional radiofrequency applications around the residual gap sites are preferred to improve PVI durability, even when complete PV disconnection is achieved.

This study has several limitations. First, the study employed a retrospective observational design; thus, the presence of selection bias pertaining to the study population cannot be ruled out. Second, given the symptomatic nature of AF and the non‐continuous monitoring, the AF recurrence rate may have been underestimated. Third, we excluded 82 patients who did not undergo the ATP challenge test from this study, which could be a source of bias. Nonetheless, we could confirm the absence of a significant difference in the baseline patient characteristics and AF recurrence rate between the study group and the excluded group, except in the sex ratio (Table S1). Fourth, we compared the AF recurrence rate between patients with FPIs in both ipsilateral PVs and those without; the results revealed disparity in the recurrence rate between the two groups (Figure S1), and the difference did not reach significance. Because AF ablation outcomes were similar between patients with FPIs in either ipsilateral PV and those with FPIs in both ipsilateral PVs in this study, a comparison of the recurrence rate between the first‐pass and non‐first‐pass groups based on first‐pass PVI achievement in at least one of the two ipsilateral PVs would be reasonable (Figure 3A). However, such disparity may change because of the differences in ablation methods and settings. Finally, this study was conducted in a single center; thus, the efficacy of FPI must be confirmed in a prospective multicenter study with a larger sample size.

## 5 CONCLUSIONS

The prevalence of spontaneous PV reconnection and DC induced by ATP was low in PVs with successful FPI. The absence of first‐pass PVI is associated with poor AF ablation outcomes, possibly owing to poor PVI quality and durability. This finding could potentially guide post‐ablation clinical decision‐making, including that pertaining to the potential early discontinuation of AAD use or reduced frequency of ambulatory monitoring, both of which affect healthcare costs and patients’ quality of life.

## CONFLICTS OF INTEREST

KI received honoraria from Johnson KK and Medtronic, Inc The other authors (YN, NT, KT, TO, YH, TO, MO, HI, KT, RN, RK, YK, AO, KI, MO, and KF) declare no conflicts of interest for this article.

## ETHICAL APPROVAL

IRB approval number: 17‐63. IRB approval date: August 21, 2017.

## Supporting information

Supplementary Material

Supplementary Material
