# Supplementary material for: Absence of first‐pass isolation is associated with poor pulmonary vein isolation durability and atrial fibrillation ablation outcomes
Source: J Arrhythm. 2021 Sep 6;37(6):1468–76. doi: 10.1002/joa3.12629 (PMC8637089; doi:10.1002/joa3.12629)
Supplement: Supplementary file 1 — Supplementary Material [file JOA3-37-1468-s002.docx]

**Table S1. Baseline patient characteristics between the study group and the excluded group**

|  | Study group | Excluded group | P value |
| --- | --- | --- | --- |
|  | n = 446 | n = 82 |  |
| Age, years | 64 ± 10 | 62 ± 11 | 0.31 |
| Male, n (%) | 370 (83) | 60 (73) | 0.04 |
| BMI, kg/m^2^ | 24.1 ± 3.5 | 24.1 ± 4.0 | 0.92 |
| CHADS2 score | 1.0 (0.0–2.0) | 1.0 (0.0–2.0) | 0.43 |
| ■ Type of AF, n (%) |  |  |  |
| Paroxysmal | 191 (43) | 31 (38) | 0.67 |
| Persistent | 175 (39) | 36 (44) |  |
| Long-standing persistent | 80 (18) | 15 (18) |  |
| ■ Comorbidities, n (%) |  |  |  |
| Heart failure | 88 (20) | 16 (20) | 0.96 |
| Hypertension | 228 (51) | 37 (45) | 0.32 |
| Diabetes mellitus | 82 (18) | 13 (16) | 0.58 |
| History of stroke | 34 (8) | 6 (7) | 0.92 |
| ■ Echocardiographic data |  |  |  |
| LAD, mm | 39.6 ± 5.9 | 39.4 ± 5.1 | 0.76 |
| LVDd, mm | 47.4 ± 5.4 | 48.3 ± 7.0 | 0.28 |
| LV ejection fraction (Teicholz), % | 63.5 ± 10.9 | 62.3 ± 13.9 | 0.44 |
| ■ Laboratory data |  |  |  |
| Hb, g/dl | 14.1 ± 1.4 | 14.2 ± 1.4 | 0.51 |
| Cre, mg/dl | 0.89 (0.75–1.00) | 0.86 (0.77–1.00) | 0.60 |
| CRP, mg/dl | 0.06 (0.03–0.13) | 0.06 (0.03–0.14) | 0.79 |
| BNP, pg/ml | 85 (40–176) | 107 (46–235) | 0.27 |

Data are expressed as mean ± standard deviation or n (%), except for the Cre, CRP and BNP, which are expressed as median (first quartile and third quartile).

BMI, body mass index; LAD, left atrial dimension; LVDd, left ventricular diastolic dimension; LV, left ventricle; Hb, hemoglobin; Cre, creatinine; CRP, C-reactive protein; BNP, brain natriuretic peptide
