# Supplementary figures and images for: Absence of first‐pass isolation is associated with poor pulmonary vein isolation durability and atrial fibrillation ablation outcomes
Source: J Arrhythm. 2021 Sep 6;37(6):1468–76. doi: 10.1002/joa3.12629 (PMC8637089; doi:10.1002/joa3.12629)

Figure S1

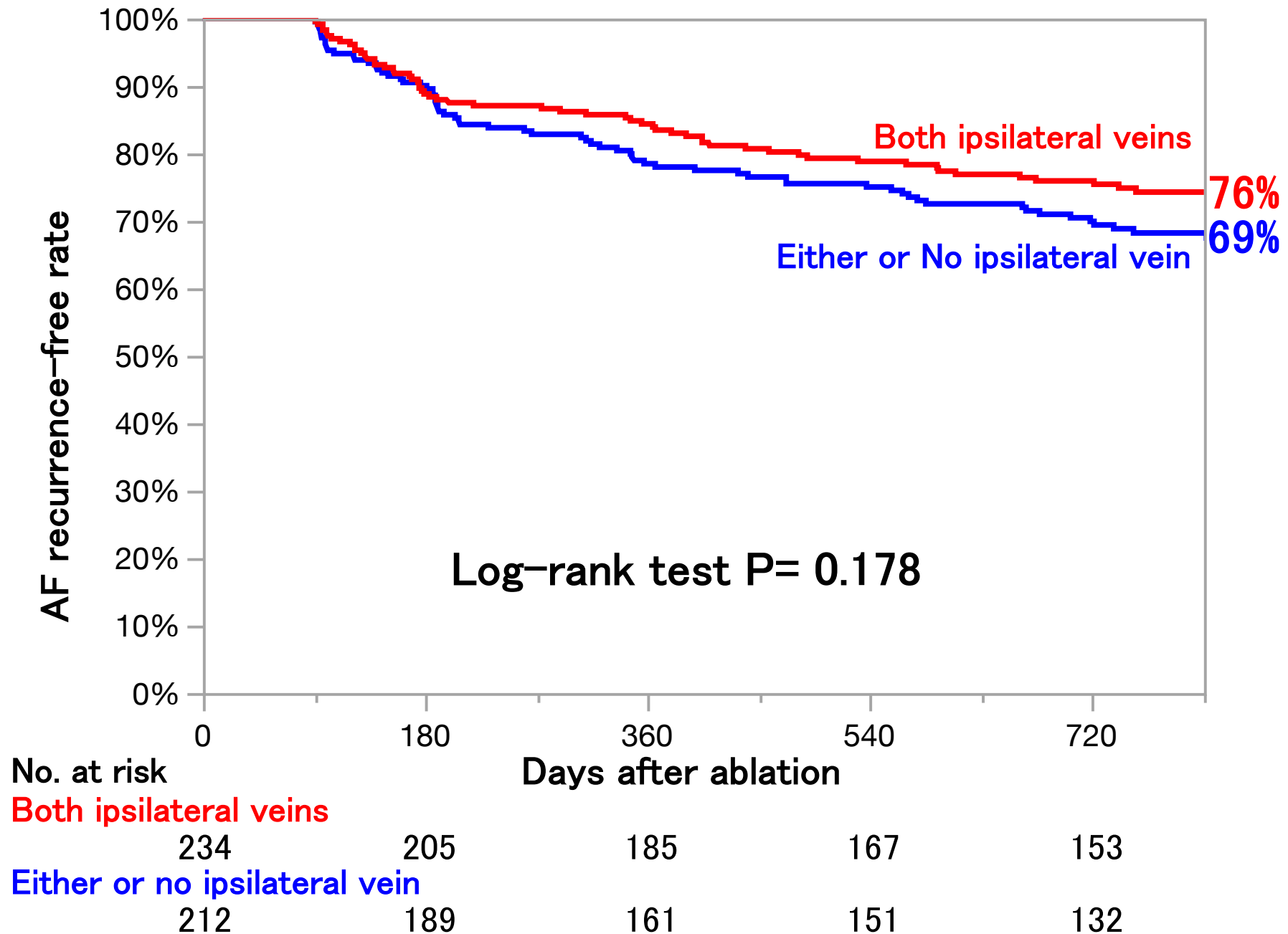

Supplement: Supplementary file 2 — Supplementary Material [file JOA3-37-1468-s001.pdf]
