# Supplementary material for: Absence of first‐pass isolation is associated with poor pulmonary vein isolation durability and atrial fibrillation ablation outcomes
Source: J Arrhythm. 2021 Sep 6;37(6):1468–76. doi: 10.1002/joa3.12629 (PMC8637089; doi:10.1002/joa3.12629)
Supplement: Supplementary file 3 [file JOA3-37-1468-s003.docx]

**Table S1**. Baseline patient characteristics between the study group and the excluded group

**Figure S1**. Both ipsilateral veins versus either ipsilateral vein or no ipsilateral vein
